# Successful Alectinib Treatment for Carcinoma of Unknown Primary with *EML4-ALK* Fusion Gene: A Case Report

**DOI:** 10.3390/curroncol28030180

**Published:** 2021-05-21

**Authors:** Keiji Sugiyama, Ai Izumika, Akari Iwakoshi, Riko Nishibori, Mariko Sato, Kazuhiro Shiraishi, Hiroyoshi Hattori, Rieko Nishimura, Chiyoe Kitagawa

**Affiliations:** 1 Department of Medical Oncology, Nagoya Medical Center, 4-1-1 Sannomaru, Naka-Ku, Nagoya 460-0001, Japan; aiai.44.tappu.77@gmail.com (A.I.); n.riko.0928@gmail.com (R.N.); peter.roos.strase20@gmail.com (M.S.); yuzushiraishi@gmail.com (K.S.); ckitag@kd6.so-net.ne.jp (C.K.); 2 Department of Pathology, Nagoya Medical Center, 4-1-1 Sannomaru, Naka-Ku, Nagoya 460-0001, Japan; akari@med.nagoya-u.ac.jp (A.I.); rnishimura-path@umin.ac.jp (R.N.); 3 Department of Clinical Genetics, Nagoya Medical Center, 4-1-1 Sannomaru, Naka-Ku, Nagoya 460-0001, Japan; hiroyoshi.hattori@nnh.go.jp

**Keywords:** carcinoma of unknown primary, next-generation sequencing, *EML4-ALK*, alectinib

## Abstract

Gene alteration in anaplastic lymphoma kinase (ALK) is rare, and the efficacy of ALK inhibitors in the treatment of carcinoma of unknown primary (CUP) with *ALK* alteration remains unclear. The patient was a 56-year-old woman who presented with cervical lymph node swelling. Computed tomography revealed paraaortic, perigastric, and cervical lymph node swelling; ascites; a liver lesion; and a left adrenal mass. A cervical lymph node biopsy was performed, and pathological diagnosis of an undifferentiated malignant tumor was conducted. Finally, the patient was diagnosed with CUP and treated with chemotherapy. To evaluate actionable mutations, we performed a multigene analysis, using a next-generation sequencer (FoundationOne^®^ CDx). It revealed that the tumor harbored an echinoderm microtubule-associated protein-like 4 (*EML4*) and *ALK* fusion gene. Additionally, immunohistochemistry confirmed ALK protein expression. Alectinib, a potent ALK inhibitor, was recommended for the patient at a molecular oncology conference at our institution. Accordingly, alectinib (600 mg/day) was administered, and the multiple lesions and symptoms rapidly diminished without apparent toxicity. The administration of alectinib continued for a period of 10 months without disease progression. Thus, ALK-tyrosine kinase inhibitors should be considered in patients with CUP harboring the *EML4-ALK* fusion gene.

## 1. Introduction

Carcinoma of unknown primary (CUP) accounts for approximately 5% of all malignancies [1,2]. Apart from a subset of patients with CUP, whose prognosis is favorable, for most patients with CUP the prognosis remains poor, despite subjection to platinum-based chemotherapy, with a median overall survival period of less than 1 year [2]. The current treatment strategy for CUP is a site-specific therapy, based on histopathological features, determined through immunohistochemistry [3,4,5], or gene expression and gene alterations, detected by next-generation sequencing (NGS). Hayashi et al. reported that genetically matched molecular targeted therapy, based on site-specific treatment evaluated by NGS, could be used to treat patients with CUP, a type of cancer, whose prognosis is unfavorable [6]. In the CUPISCO trial, anaplastic lymphoma kinase (*ALK*) rearrangement was detected in 0.7% of the cases [7]. Although *ALK* is recognized as a driver oncogene in non-small-cell lung cancer (NSCLC), and anti-ALK therapy is the standard of care provided to NSCLC patients with *ALK* rearrangement [8,9,10], the efficacy of ALK-tyrosine kinase inhibitors (TKIs) for CUP with *ALK* rearrangement remains unclear.

## 2. Case Presentation

**Figure 1:**
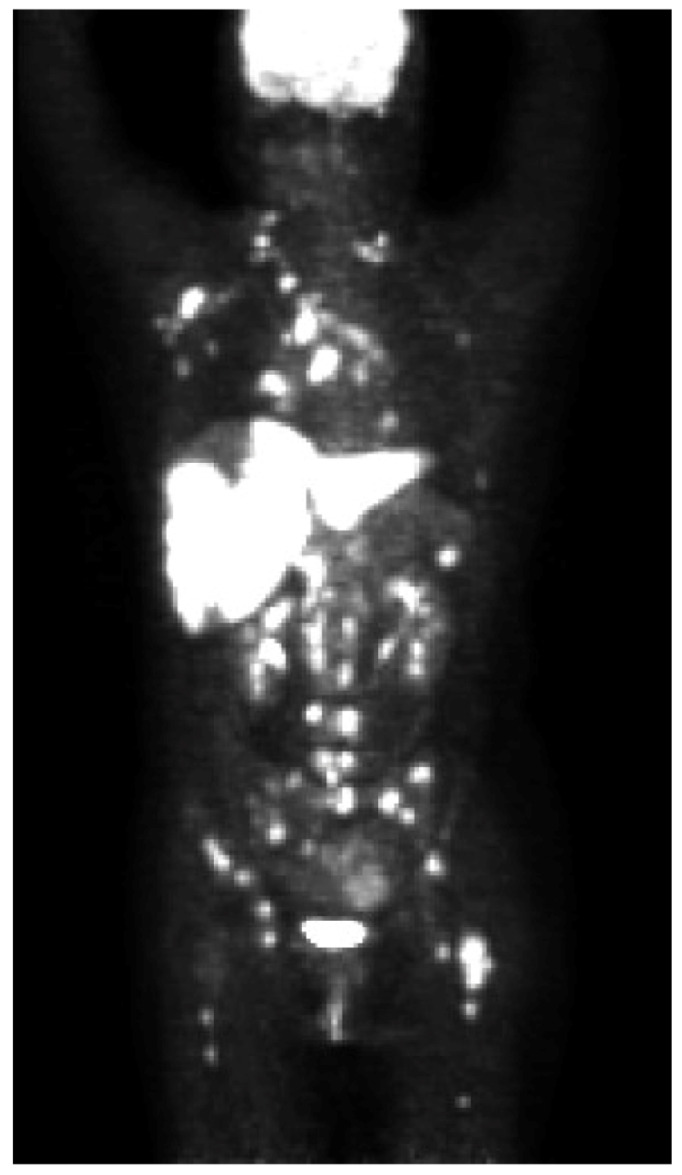
FDG-PET/CT performed before treatment. The figure shows FDG uptake in multiple lesions (liver, bone, and lymph node lesions). FDG-PET/CT: fluorine-18-2-deoxy-d-glucose positron emission tomography and computed tomography.

**Figure 2:**
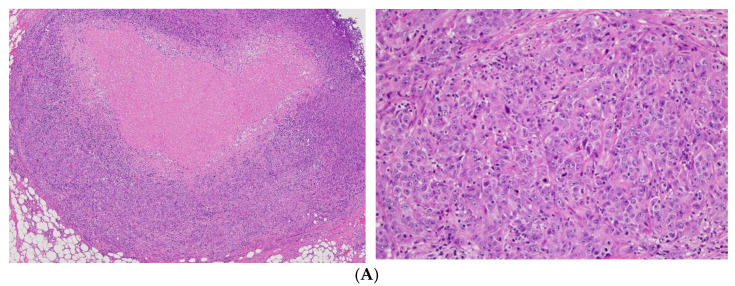

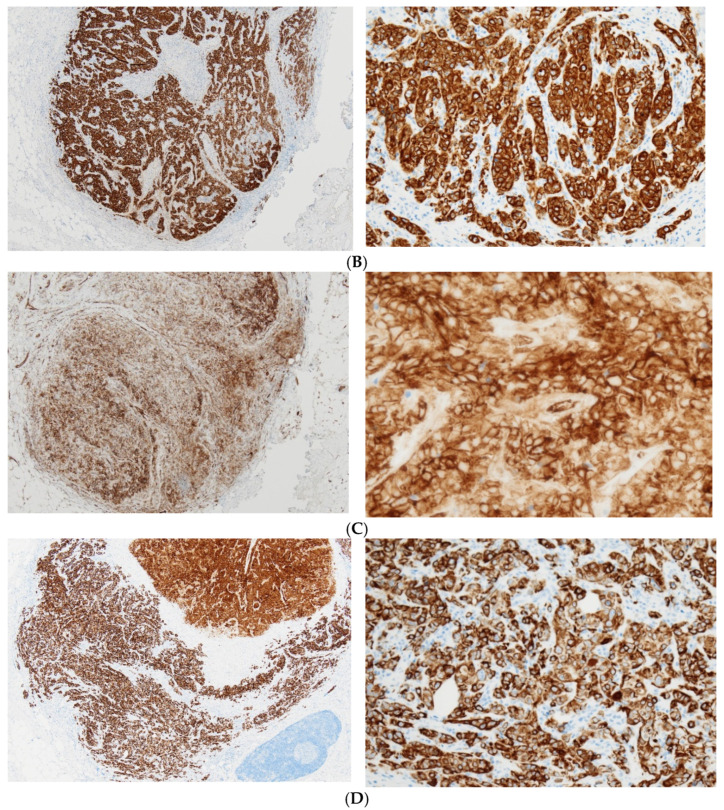
Hematoxylin-eosin (HE) staining and immunohistochemical (IHC) features of the tumor. HE staining and IHC features of AE1.3, CD31, and CK7 are shown. (**A**) HE staining (left: X4 objective, right: X40 objective); (**B**) AE1.3 (left: X4 objective, right: X20 objective); (**C**) CD31 (left: X4 objective, right: X40 objective); (**D**) CK7 (left: X4 objective, right: X20 objective). Immunopositivity for AE1.3, CD31, and CK7 is evident.

**Figure 3:**
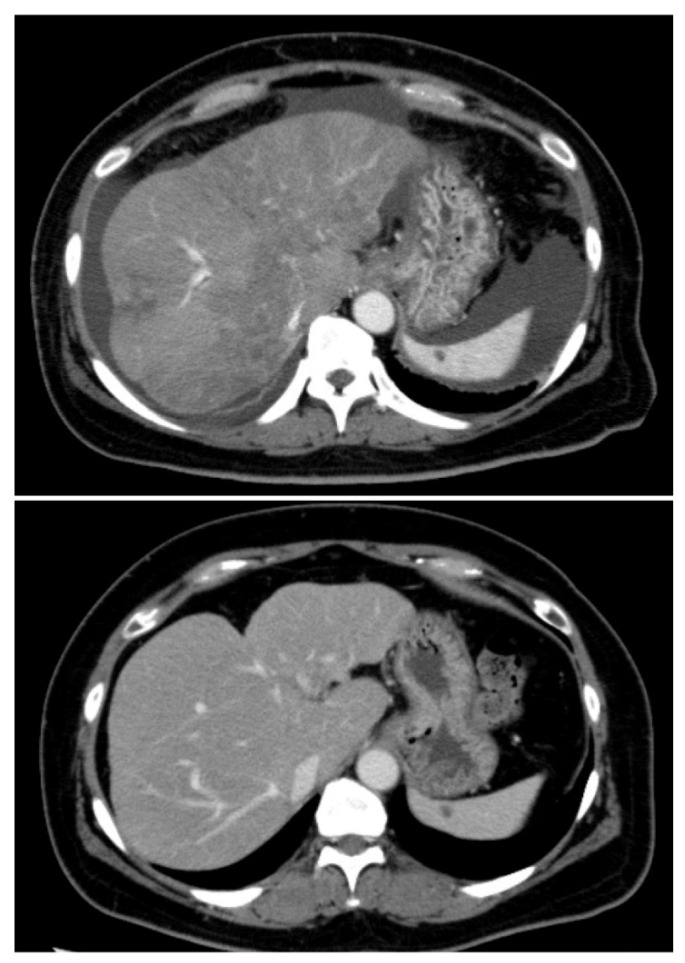
Computed tomography performed before (**above**) and after (**below**) alectinib therapy. Liver lesion and ascites disappeared after alectinib therapy.

**Figure 4:**
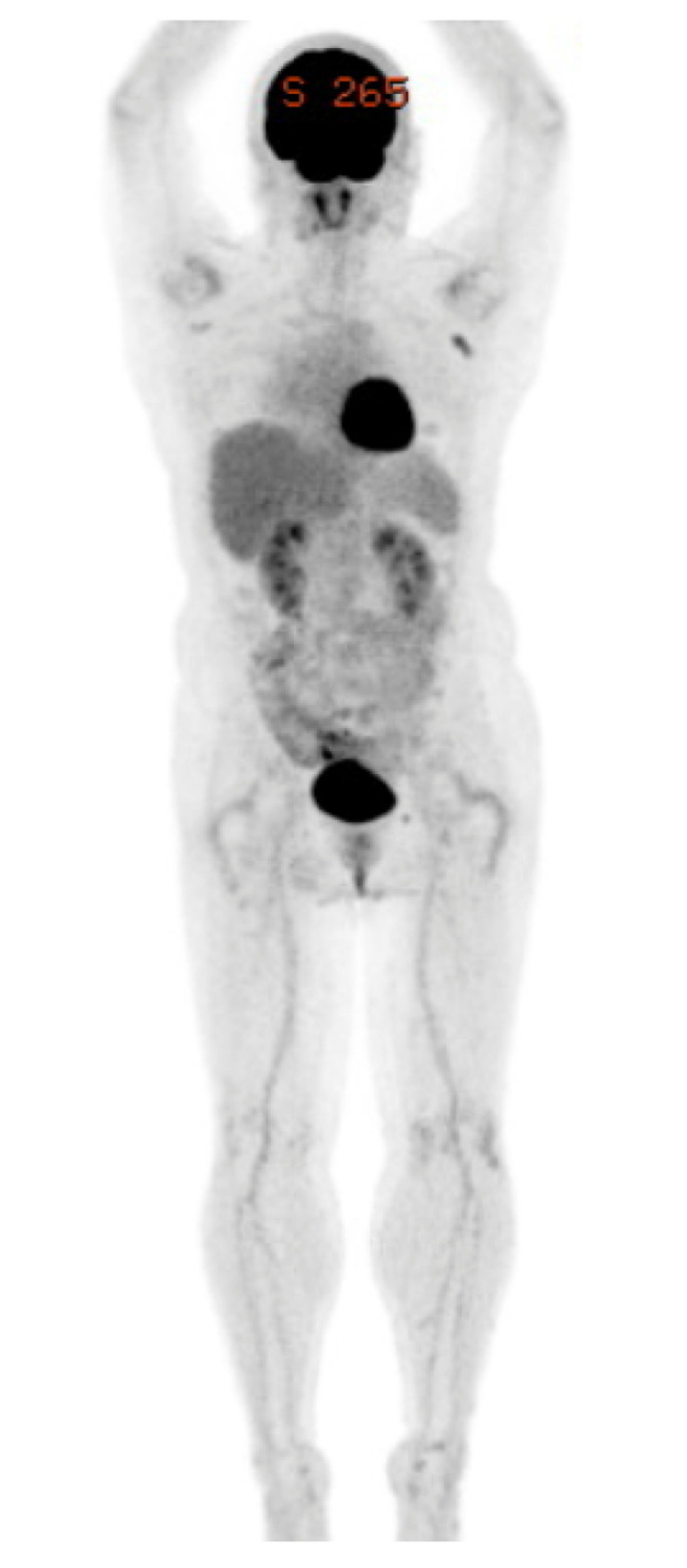
FDG-PET/CT after alectinib therapy. FDG uptake in the multiple lesions was normal. FDG-PET/CT: fluorine-18-2-deoxy-d-glucose positron emission tomography and computed tomography.

A 56-year-old woman complained of cervical lymph node swelling. Contrast-enhanced computed tomography (CT) revealed the presence of swelling of multiple lymph nodes (paraaortic, perigastric, and cervical), a liver lesion, a left adrenal mass, and ascites. To identify the primary tumor site, fluorine-18-2-deoxy-d-glucose positron emission tomography/CT (FDG-PET/CT) was performed; however, the primary site could not be detected (Figure 1). A cervical lymph node biopsy was performed, and based on the findings, a diagnosis of an undifferentiated malignant tumor was conducted. Immunohistochemical evaluation (positivity for cytokeratin AE1.3, cluster of differentiation [CD] 31, and cytokeratin7 and negativity for cytokeratin20, thyroid transcription factor-1 [TTF-1], napsin-A, p40, p63, CDX2, PAX8, and estrogen receptor) did not indicate any differentiation or could not identify the primary tumor site (Figure 2). The levels of serum lactate dehydrogenase (893 IU/L), carcinoma antigen (CA)-125 (5645 IU/L), and CA15-3 (1375 IU/L) increased, but those of carcinoembryonic antigen (CEA) and CA19-9 did not. Colonoscopy revealed a small lesion in the sigmoid colon. The pathological diagnosis of the colon lesion was a well-differentiated adenocarcinoma, and an immunohistochemical examination showed a staining pattern, typical of colorectal adenocarcinoma (cytokeratin20 and CDX2 positivity and cytokeratin7 negativity). As the tumor cells of the cervical lymph node specimen were CD31-positive, we considered the possibility of epithelioid angiosarcoma (EA). However, the cells showed negative results for CD34 and factor VIII, and EA was ruled out after consultation with a specialist in soft-tissue pathology. Finally, this patient was diagnosed with CUP with an unfavorable subset and early-stage colorectal adenocarcinoma. The tumor was predominantly located (multiple liver lesions, ascites, and peritoneal carcinomatosis) under the diaphragm space. Thus, we initially assumed that the cancer originated in the gastrointestinal tract and chose fluorouracil, oxaliplatin, and leucovorin (FOLFOX) as the first-line therapy [11]. The patient responded to FOLFOX, showing a decrease in CA125 and CA15-3 levels; the duration of response was 3 months. To evaluate the potential of molecular targeted therapy, we performed a genetic evaluation using FoundationOne^®^ CDx for a cervical lymph node sample. The test was completed successfully and revealed significant findings with *EML4-ALK* fusion gene expression. Apart from the *EML4-ALK* fusion gene, actionable mutation and remarkable findings, including high microsatellite instability and high tumor mutational burden, were not detected. Immunohistochemistry confirmed ALK protein expression in tumor cells. In the molecular oncology conference at our institute, ALK inhibitors were recommended for the patient’s treatment. At the beginning of alectinib therapy (600 mg/day), the patient complained of tumor-associated symptoms, including bone and abdominal pain, anorexia, and malaise. After subjection to alectinib therapy, the patient’s symptoms alleviated, and the tumor marker levels declined markedly (Figure 3). PET-CT performed 6 months after the treatment showed that a complete metabolic response was achieved, except for reactive lymphadenopathy due to dermatitis in the left axillary lymph node (Figure 4). Alectinib therapy was continued for more than 1 year without disease progression or severe adverse events.

## 3. Discussion

Herein, we have presented the case of a patient with CUP, harboring *EML4-ALK* fusion gene; expression of the gene was detected by NGS and the patient was treated with alectinib. Some retrospective and prospective studies have demonstrated the role of NGS in the determination of genetically matched molecular targeted therapy for CUP. In a comprehensive analysis conducted by the Memorial Sloan Kettering Cancer Center, NGS was used for 150 patients diagnosed with CUP; 30% of the patients presented with potentially targetable genomic alterations, and 10% received targeted therapies. Furthermore, several patients with *ERBB2* amplification, *BRAFV600E* mutation, *KIF5B-ALK* fusion, *NCOA4-RET* fusion, *FGFR2/3* fusion, and *IRF2BP-NTRK1* fusion showed a response to matched specific kinase inhibitors [12]. In a trial conducted by Hayashi et al., precision therapy, based on NGS (molecular targeted therapy), was performed for CUP [6]. *EGFR* mutations (E709G, L861Q, R776C, and G719C) were detected in five of the ninety-seven patients in their trial. Four of the five patients received afatinib, an EGFR TKI, and two of them achieved a response with subjection to more than 6 months of treatment. Overall, NGS-based targeted therapy provides considerable clinical benefit to a limited, molecularly selected subset of patients with CUP.

The *EML4-ALK* fusion gene is recognized as the second most common driver gene mutation occurring in lung adenocarcinoma, and ALK inhibitors have shown marked efficacy compared with conventional chemotherapy in affected patients. It was crucial to determine whether CUP or lung adenocarcinoma with *ALK* rearrangement was present in our patient. Previous studies have suggested that lung cancers with *EML4-ALK* fusion are characterized as adenocarcinoma with TTF-1 expression based on the immunohistochemistry results, and this rearrangement has been noted in lung cancers of the TTF-1 cell lineage [13]. Our patient showed negative results for TTF-1, a marker for lung adenocarcinoma, and CT did not show an apparent lung nodule. Hence, the possibility of TTF-1–positive lung adenocarcinoma was extremely low. Additionally, the presence of *EML4-ALK* fusion gene is not an abnormality specific to lung cancer; it has also been reported in colorectal cancer and CUP [7,14]. An increasing number of reports have shown the efficacy of ALK inhibitors in patients with advanced cancers other than lung cancer with *ALK* abnormalities [15,16]. Therefore, *ALK* rearrangement seems to be a tumor agnostic abnormality across organs and merits active screening in patients with advanced cancer.

## Data Availability

As this is a case report, no data analysis has been performed.
